# Optimized methods to image hepatic lipid droplets in zebrafish larvae

**DOI:** 10.1242/dmm.050786

**Published:** 2024-11-25

**Authors:** Nouf Khan, Talhah Mohd Salmi, Anthony P. Karamalakis, Anjana Ramdas Nair, Kirsten C. Sadler, Andrew G. Cox

**Affiliations:** ^1^ Program in Biology, New York University Abu Dhabi, PO Box 129188, Abu Dhabi, United Arab Emirates; ^2^ Peter MacCallum Cancer Centre, Melbourne, VIC 3000, Australia; ^3^ The Sir Peter MacCallum Department of Oncology, The University of Melbourne, Melbourne, VIC 3000, Australia; ^4^ Department of Biochemistry and Pharmacology, The University of Melbourne, Melbourne, VIC 3000, Australia

**Keywords:** Lipid droplet, Liver disease, Steatosis, Zebrafish

## Abstract

The optical transparency of zebrafish larvae enables visualization of subcellular structures in intact organs, and these vertebrates are widely used to study lipid biology and liver disease. Lipid droplet (LD) presence is a prevalent feature of healthy cells, but, under conditions such as nutrient excess, toxicant exposure or metabolic imbalance, LD accumulation in hepatocytes can be a harbinger of more severe forms of liver disease. We undertook a comprehensive analysis of approaches useful to investigate LD distribution and dynamics in physiological and pathological conditions in the liver of zebrafish larvae. This comparative analysis of the lipid dyes Oil Red O, Nile Red, LipidTox and LipidSpot, as well as transgenic LD reporters that rely on EGFP fusions of the LD-decorating protein perilipin 2 (PLIN2), demonstrates the strengths and limitations of each approach. These protocols are amenable to detection methods ranging from low-resolution stereomicroscopy to confocal imaging, which enables measurements of hepatic LD size, number and dynamics at cellular resolution in live and fixed animals. This resource will benefit investigators studying LD biology in zebrafish disease models.

## Teaser

**Editor's choice:** A comprehensive comparative analysis of tools and approaches to study lipid droplet dynamics in the zebrafish larval liver under physiological and pathological conditions.

## INTRODUCTION

The liver performs critical functions that play a key role in maintaining metabolic homeostasis and blood detoxification in vertebrates (Gordillo et al., 2015; Miyajima et al., 2014). Hepatocytes regulate numerous metabolic processes in the liver, including glucose and lipid metabolism. In fed state, hepatocytes exhibit the profound capacity to store energy in the form of glycogen granules and lipid droplets (LDs) (Han et al., 2016; Petersen et al., 2017; Shimano and Sato, 2017). In humans, accumulation of LDs in hepatocytes (steatosis) is most frequently caused by obesity, diabetes, overconsumption of alcohol, fasting or toxicant exposure. Steatosis can resolve without any apparent long-term consequence, but, in cases in which the cause of steatosis persists, steatosis can progress to a more pathological state known as metabolic dysfunction-associated steatotic liver disease (MASLD) or alcohol-related liver disease (ALD) (Bondini et al., 2007; O'Shea et al., 2010; Rinella et al., 2024). Additionally, simple steatosis can synergize with other stressors to generate a more severe form of disease. Importantly, the same conditions cause MASLD and ALD in rodents and zebrafish. The molecular and cellular mechanisms that underlie the initiation and progression of fatty liver are largely conserved across vertebrates (Bertola, 2018; Chang et al., 2023; Goessling and Sadler, 2015), making these models useful to understand the pathophysiology of this disease.

Lipids are important in cellular processes such as membrane biogenesis, cellular energetics and signalling (Broadfield et al., 2021). Cytoplasmic LDs are organelles with a monophospholipid membrane encompassing a hydrophobic core comprised of triglycerides and sterol esters (Olzmann and Carvalho, 2019; Zadoorian et al., 2023). They can be coated with structural proteins, including perilipins (PLINs), which regulate their size, metabolism and interaction with other organelles (Kimmel and Sztalryd, 2016). LDs play a key role in energy storage (Mejhert et al., 2022; Olzmann and Carvalho, 2019; Tauchi-Sato et al., 2002; Wilfling et al., 2013) and, as dynamic organelles, can undergo anabolic expansion (lipogenesis) or catabolic breakdown (lipolysis and lipophagy) (Fujimoto et al., 2007; Kuerschner et al., 2008; Kurat et al., 2009; Olzmann and Carvalho, 2019; Roberts and Olzmann, 2020; Zadoorian et al., 2023). Dysregulation of LD homeostasis is closely linked to hepatic steatosis (Carr and Ahima, 2016). The size, composition and properties of LDs vary with disease type, with MASLD and ALD characterized by predominantly large LDs (i.e. macrovesicular), and some other less common causes of LD accumulation presenting with smaller LDs. In addition to the causative role that LDs play in obesity, MASLD and ALD, dysregulation of LDs has been implicated in neurodegenerative diseases and cancer (Cruz et al., 2020; Gluchowski et al., 2017; Maan et al., 2018; Pereira-Dutra et al., 2019; Seebacher et al., 2020; Walther and Farese, 2012). Therefore, tools to monitor, assess and perturb LDs in disease models can provide insights into the role of LDs in pathophysiology.

Studying LD dynamics using whole-animal *in vivo* models is crucial to understand how these organelles form and contribute to liver disease. However, observing LD dynamics *in situ* is extremely challenging in rodent models as the tissue must be removed from the animal for examination. In contrast, transparent zebrafish larvae are amenable to whole-animal imaging of LDs under both live and fixed conditions using simple and advanced microscopy approaches (Patton et al., 2021; Salmi et al., 2019). Elegant imaging studies in zebrafish using timelapse microscopy and 3D reconstruction of subcellular organelles have uncovered new information about developmental and pathological processes (Salmi et al., 2019). In particular, lipid dyes have been used in screens to identify genetic and pharmacological regulators of lipid metabolism in zebrafish (Clifton et al., 2010; Farber et al., 2001; Saele et al., 2018). The high conservation of the molecular players and cellular processes that give rise to disease make zebrafish an excellent model system to complement studies using mammalian models (Goessling and Sadler, 2015; Lieschke and Currie, 2007). Moreover, the large sample sizes available when studying zebrafish embryos and larvae offer unique opportunities to observe the natural variability across a population of animals and to perform large-scale screens with high statistical power at a fraction of the cost of mammalian models (Cox and Goessling, 2015).

Hepatocytes in the developing zebrafish liver are mature and able to metabolize lipid by 5 days post fertilization (dpf) (Cox and Goessling, 2015; Farber et al., 2001; Goessling and Sadler, 2015). Therefore, using larvae between 4 and 6 dpf for studies on hepatic lipid metabolism provides a controlled environment with uniform nutrient delivery from the yolk. At this stage, the larvae are small and transparent, allowing the integration of cutting-edge imaging technology with mechanistic studies of steatosis (Cinaroglu et al., 2011; Freeburg et al., 2024; Howarth et al., 2011; Passeri et al., 2009; Sadler et al., 2005; Salmi et al., 2019). We and others have developed genetic, nutritional and toxicant-induced models of steatosis using zebrafish larvae (Amali et al., 2006; Bambino et al., 2018; Chang et al., 2023; Cinaroglu et al., 2011; Howarth et al., 2011; Matthews et al., 2009; Passeri et al., 2009; Pozo-Morales et al., 2024; Sapp et al., 2014; Vaidyanathan et al., 2022). However, the approaches to studying lipids in zebrafish have not yet been standardized or compared for their relative utility.

Here, we performed a comparative study of multiple approaches available to label and image hepatic LDs in live and fixed zebrafish larvae. We present data on the efficacy of staining with a range of dyes including Oil Red O (ORO), Nile Red (NR), LipidTox and LipidSpot. We compared these approaches with a transgenic LD reporter, *Tg(fabp10a:EGFP-PLIN2)*, using which LD dynamics can be imaged in a temporal manner. Importantly, we present a detailed description of the approaches used to prepare larvae and quantitatively analysed the incidence of steatosis in a population, as well as measured the number and size of LDs using live and fixed samples. We anticipate that these methods will be of great utility for future studies on hepatic steatosis or relevant metabolic diseases that utilize the zebrafish as an *in vivo* model.

## RESULTS

### Comparative approaches for detecting LDs in zebrafish livers

**Fig. 1.**
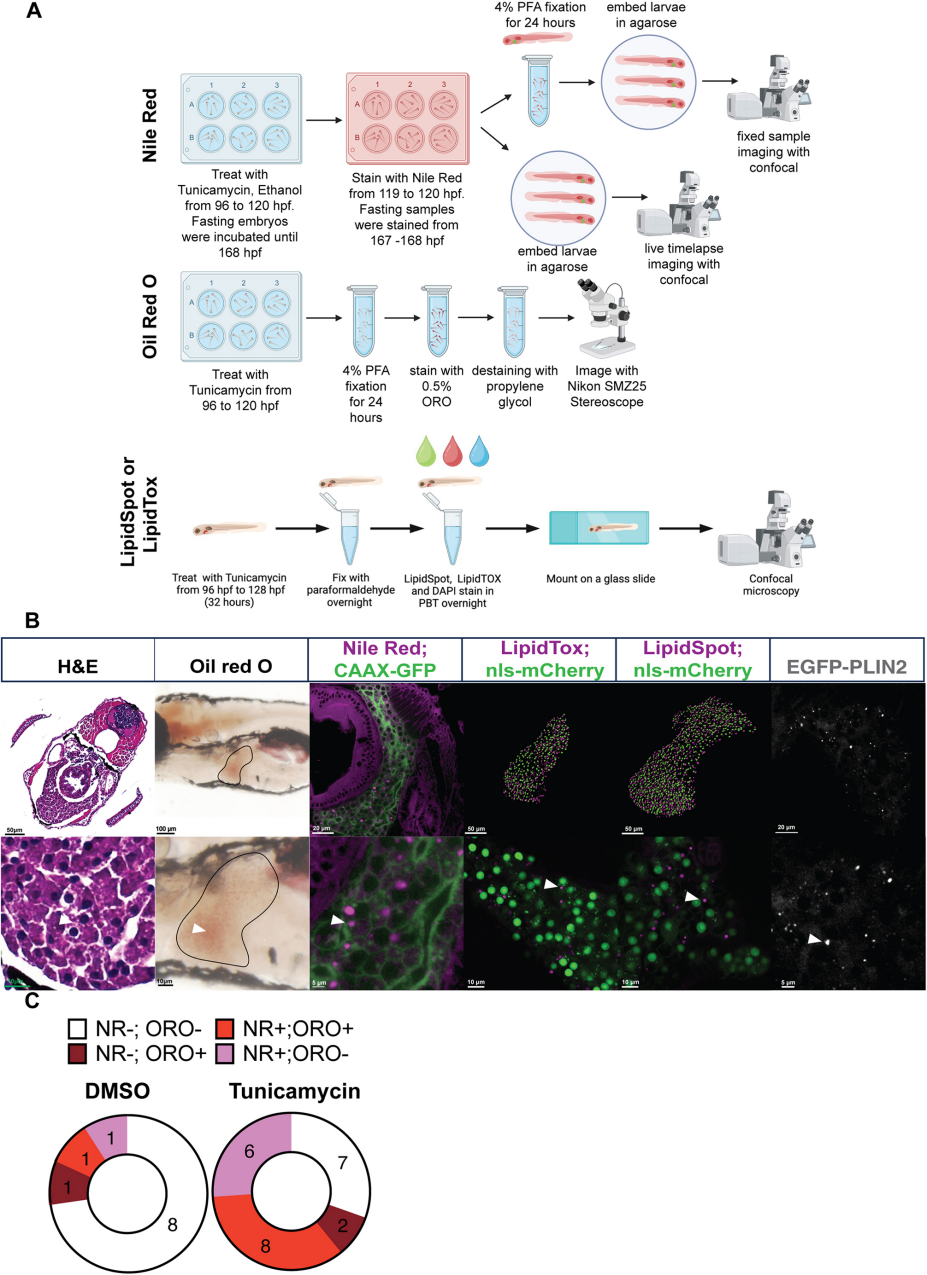
**Toolbox to assess hepatic lipid droplets (LDs) in zebrafish larvae*.*** (A) Treatment protocol for inducing steatosis in zebrafish embryos with either tunicamycin (TM) or fasting to 168 h post fertilization (hpf) [7 days post fertilization (dpf)] to assess the use of Oil Red O (ORO), Nile Red (NR), LipidTox and LipidSpot to detect LDs. DAPI, 4′,6-diamidino-2-phenylindole; PBT, phosphate buffered saline containing Tween 20; PFA, paraformaldehyde. (B) Comparison of methods used to detect LD accumulation in the liver of 5 dpf larvae following treatment with TM. Histological [Haematoxylin and Eosin (H&E)] assessment of steatosis compared to dye-based evaluation of LD staining as outlined in A. Staining with ORO, NR, LipidSpot, LipidTox and the transgenic line *Tg(fabp10a:EGFP-PLIN2)* shows LDs in hepatocytes as viewed by light stereomicroscopy (ORO), multiphoton microscopy (LipidTox and LipidSpot, Imaris rendering of 3D-confocal *z*-stacks) or confocal microscopy (NR and EGFP-PLIN2, single *z*-section). LipidTox and LipidSpot samples were imaged along with a transgenic marker for the nucleus *Tg(fabp10a:NLS-mCherry)*, and the NR images were obtained in larvae with EGFP targeted to the hepatocyte cell membrane [*Tg(fabp10a:CAAX-EGFP)*]. Scale bars: 5, 10, 20, 50 and 100 µm as indicated on each image individually. The images show a typical liver with LD accumulation detected by each staining method as viewed from over 100 embryos from over 20 clutches per staining method. (C) NR is more sensitive than ORO at detecting LD presence in hepatocytes. Larvae were treated with 0.5 µg/ml TM or dimethyl sulfoxide (DMSO) from 96 to 120 hpf, collected and stained with NR, imaged using confocal microscopy, scored as negative or positive for steatosis, then recovered as individual larvae and processed for ORO staining, and scored as negative or positive for steatosis based on the presence of ORO-labelled LDs. Each larva was categorized based on the steatosis score for both dyes. The experiment was carried out on a total of 34 larvae from two clutches.

There are multiple tools to detect LDs using imaging-based approaches in live and fixed zebrafish larvae. Previous work to detect LDs in zebrafish used the dyes ORO (Cinaroglu et al., 2011; Howarth et al., 2013), LipidTox (Vaidyanathan et al., 2022), NR (Flynn et al., 2009; Freeburg et al., 2024; Le Mentec et al., 2023; Pozo-Morales et al., 2024), BODIPY-labelled fatty acids (Farber et al., 1999, 2001) and EGFP-tagged Perilipins (Lumaquin et al., 2021; Wilson et al., 2021). We compared the performance of these tools and assessed LipidSpot as a new dye-based method to detect LDs in live and fixed zebrafish larval livers at 5 dpf (Fig. 1A). We induced steatosis using the well-established toxin tunicamycin (TM), which blocks protein N-glycosylation, causing endoplasmic reticulum stress, and leads to LD accumulation by a mechanism that is not yet understood (Cinaroglu et al., 2011; Feng et al., 2017; Howarth et al., 2013). This allowed a side-by-side comparison of each approach for the utility in detecting hepatic LDs (Fig. 1A).

In the clinical setting, steatosis is typically scored by inspection of Haematoxylin and Eosin (H&E)-stained sections. Although histopathological review of liver sections in the experimental setting reveals some extent of steatosis, it lacks sensitivity (Fig. 1B). In contrast, neutral lipid dyes are effective at staining LDs by virtue of their extremely high affinity for neutral lipids such as triacylglycerides. ORO is a colorimetric neutral lipid stain that can be used to detect LDs on cryosections or in whole-mount specimens using light microscopy (Fig. 1B). Fluorescent neutral lipid dyes, such as NR, LipidTox and Lipid Spot, can be used for whole-mount imaging by fluorescent confocal microscopy in live or fixed samples. The fluorescent dyes allow for high-resolution analysis of LD properties and dynamics with subcellular resolution (Fig. 1B). Fluorescent transgenes provide a stable method for identifying intracellular structures, and these can be combined with fluorescent dyes (Fig. 1B). Transgenic lines expressing EGFP-PLIN2 have been shown to effectively label LDs in zebrafish enterocytes and hepatocytes (Wilson et al., 2021) and were included here (Fig. 1B).

We found that each of these tools can be used to detect LDs in hepatocytes of larvae exposed to 0.5 µg/ml TM (Fig. 1B). ORO has the advantage that it is inexpensive and stable, and samples can be visualized using brightfield microscopy on a simple stereoscope, allowing screening of hundreds of larvae in a few hours. We typically stained 15-30 larvae per sample with ORO, and these could be quickly scored as positive or negative for LD accumulation in the liver using a brightfield stereoscope, enabling population-scale assessment of steatosis incidence. However, the staining could be variable, and background staining, pigment or the presence of yolk can obscure the liver. Owing to the background staining, we set a threshold of three clearly identifiable LDs in a liver to assign a positive score. Moreover, ORO is highly sensitive to de-staining, and this can introduce variability. In contrast, NR appears to be more specific and less subject to misidentification of LDs, and we therefore set a threshold of two LDs in a *z*-stack (which may not go through the entire liver) to assign a positive score. We compared the sensitivity of the method using ORO to that using NR for detecting LD accumulation in the liver in the same animals. Larvae treated from 96 to 120 h post fertilization (hpf) with TM or dimethyl sulfoxide (DMSO) were first imaged live using the NR staining protocol outlined in Fig. 1A, and were scored for the presence or absence of steatosis based on the observation of two or more LDs in a *z*-stack through at least 30% of the left liver lobe. Each larva was then removed from the agarose gel, individually fixed, processed for ORO staining as outlined in Fig. 1A, and scored for the presence or absence of steatosis. By comparing these staining methods on 31 individual larvae from two clutches (11 DMSO treated, 21 TM treated), we found that 24 were scored as positive in both assays, whereas six were scored as positive by NR but not by ORO, and three were scored as positive by ORO but not by NR. This translated to a steatosis incidence in TM-treated larvae of 78% based on NR staining compared to 56% based on ORO staining (Fig. 1C). This indicates that NR is more sensitive than ORO at detecting steatosis.

All fluorescent lipid markers analysed robustly labelled LDs in hepatocytes, providing a bright signal that can be quantified to assess steatosis incidence measurement in a population, and can also be scored for intensity, distribution and size of LDs. In comparison, the transgenic LD reporter *Tg(fabp10a:EGFP-PLIN2)* was dimmer than the dye-based approaches when detecting hepatic LDs (Fig. 1B). We have thus defined a toolkit of approaches that enable analysis of LD accumulation in hepatocytes, each with distinct advantages and caveats (Table 2). NR faintly labelled cell membranes, whereas LipidTox and LipidSpot had minimal background staining. Although background staining can present a challenge for scoring and quantification, it has the advantage of highlighting the liver architecture. NR can label both LDs and phospholipids, and the detection of these different pools of cellular lipids can be discriminated using different excitation/emission settings (Table 1) (Brown et al., 1992; Greenspan et al., 1985). We examined NR-stained fixed larvae that had fasting-induced steatosis with an excitation wavelength of 450-500 nm/>528 nm emission to identify cytoplasmic LDs only, compared to an excitation wavelength of 515-560 nm/>590 nm emission, which can capture both LDs and phospholipids (Brown et al., 1992; Greenspan et al., 1985). We found that although there was less background in the 450-500/>528 nm samples, both conditions identified the same LDs (Fig. S1). The additional benefit of detecting the tissue architecture using the longer wavelengths made this preferable for our studies.

**Table 1.**
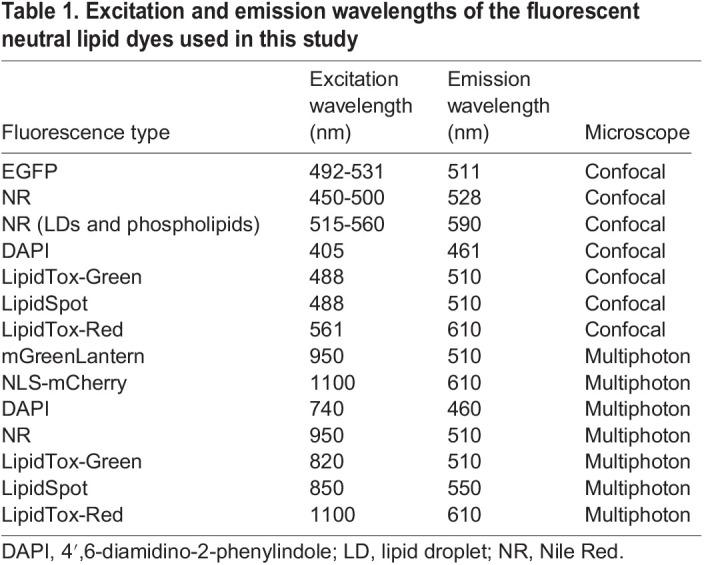
Excitation and emission wavelengths of the fluorescent neutral lipid dyes used in this study

**Table 2.**
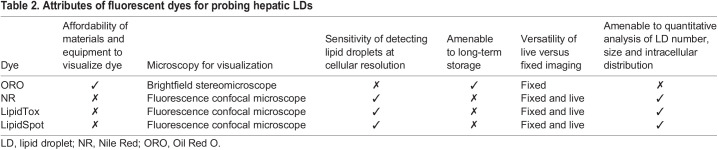
Attributes of fluorescent dyes for probing hepatic LDs

### NR as a tool to assess steatosis incidence and severity in fixed and live zebrafish larvae

**Fig. 2.**
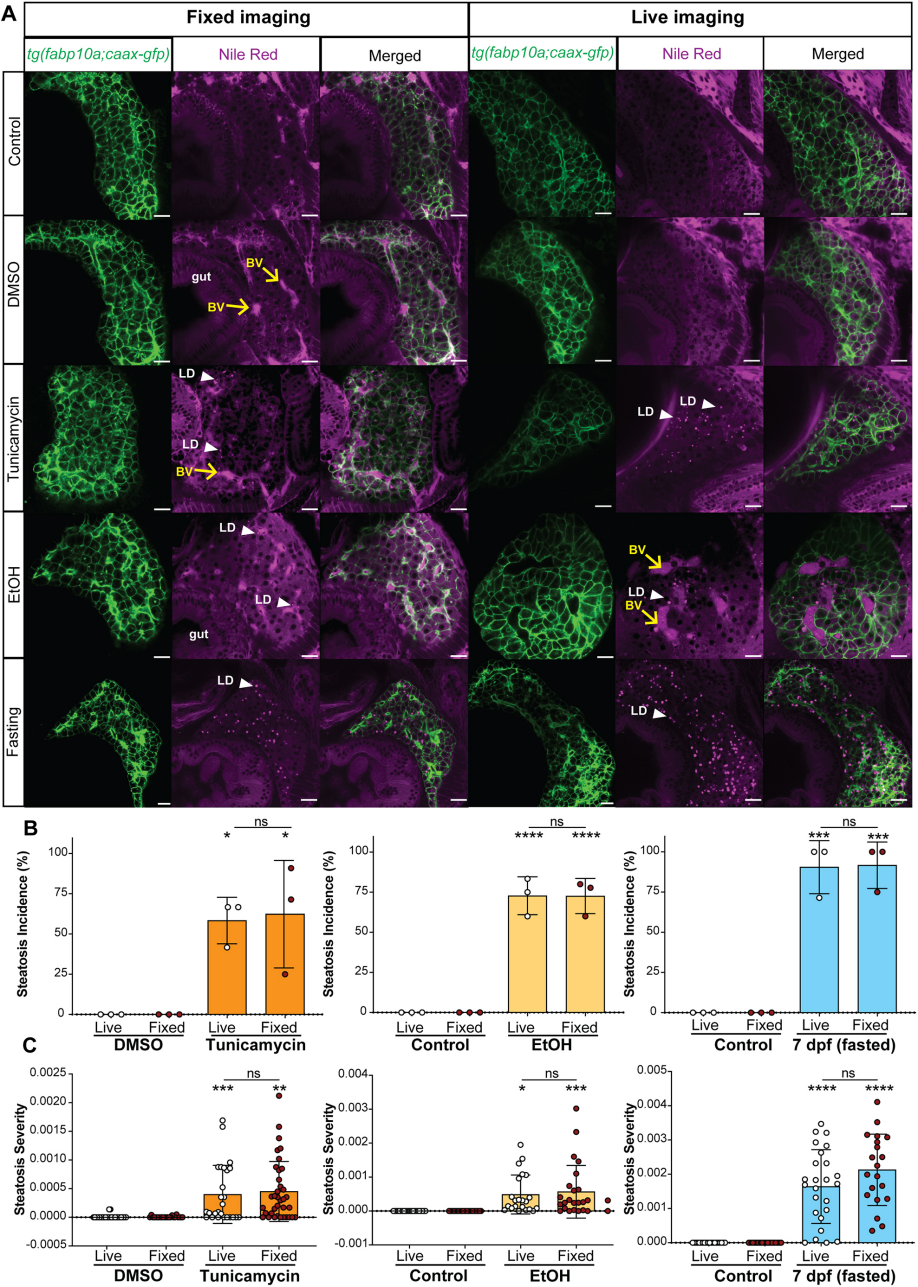
**NR staining of LDs in live and fixed larvae shows increased steatosis incidence and severity in response to TM, ethanol and fasting.** (A) Representative confocal images of hepatocytes (EGFP membrane marker; green) and LDs (magenta) in 120 hpf zebrafish untreated or treated with DMSO (vehicle), 350 mM ethanol (EtOH), 0.5 μg/ml TM from 96 to 120 hpf, and fasted larvae (7 dpf) stained with NR. The transgenic line *Tg(fabp10a:CAAX-EGFP)* was used to mark hepatocyte membrane. Scale bars: 20 μm. White arrowheads (LD) point to examples of LDs and yellow arrows (BV) point to blood vessels. Images are representative of 30 larvae from three clutches. (B) Steatosis incidence measured by percentage of samples having two or more LDs in a single *z*-plane through the middle of the liver (ns, not significant; *P<0.05, ***P<0.001, ****P<0.0001 by unpaired two-tailed Student's *t*-test, *n*=3 clutches, 30 larvae). (C) Steatosis severity quantified by the number of LDs divided by the total area of the liver in a single *z*-plane for each treatment condition (ns, non-significant; *P<0.05, **P<0.01, ***P<0.001, ****P<0.0001 by unpaired two-tailed Student's *t*-test, *n*=3 clutches, 30 larvae).

Live imaging using zebrafish larvae permits the visualization of cellular structures in developing or diseased organs *in vivo* (Goessling and Sadler, 2015; Patton et al., 2021; Salmi et al., 2019). To examine the LDs at cellular resolution in fixed samples, we optimized a fluorescent microscopy-based method using NR, which utilized a transgenic line that expresses eGFP targeted to hepatocyte cell membrane [*Tg(fabp10a:CAAX-EGFP)*] (Nussbaum et al., 2013) to mark hepatocytes. There were no LDs in control or DMSO-treated larvae (Fig. 2A), but LDs were detected in hepatocytes caused by multiple interventions that induced steatosis: endoplasmic reticulum stress caused by TM, ethanol exposure as a model of ALD, and fasting to 7 dpf, with fasting having the most robust LDs (Fig. 2A) (Pozo-Morales et al., 2024). We compared the performance of NR in live and fixed samples, which showed that LD detection was comparable with both methods (Fig. 2A). This illustrates that NR can be used in experiments to quantify LD size, distribution and number in fixed samples and can also be used for assessing LD dynamics in live samples. NR also detected lipid in the blood vessels and the adjacent gut, providing a means to assess larval anatomy and hepatic architecture in the same images (Fig. 2A). This feature revealed that blood vessels appear focally dilated in ethanol-exposed samples (Fig. 2A).

We observed that not only did more larvae in the treated population have evidence of LDs in hepatocytes, which was scored as steatosis incidence (Fig. 2B), but also that the number of LDs varied across animals, which we developed a method to assess. Incidence of steatosis was assessed by manually counting the number of larvae that displayed more than two LDs per liver (Fig. 2B), similar to what was reported by others (Le Mentec et al., 2023). This showed that all treatments significantly increased the incidence of steatosis from 50% in TM-exposed larvae to 100% in fasted larvae, and there was no significant difference in the incidence as measured in fixed or live samples. To quantify the degree of LD accumulation in each sample, the number of LDs was divided by the surface area of the liver, with the ratio used as a steatosis severity score (Fig. 2C). The steatosis severity in control larvae was zero, and all exposures generated significantly higher steatosis severity, with the most severe steatosis observed in fasted samples. There was no significant difference between live and fixed samples (Fig. 2C), providing flexibility for using NR to assess LDs in zebrafish.

**Fig. 3.**
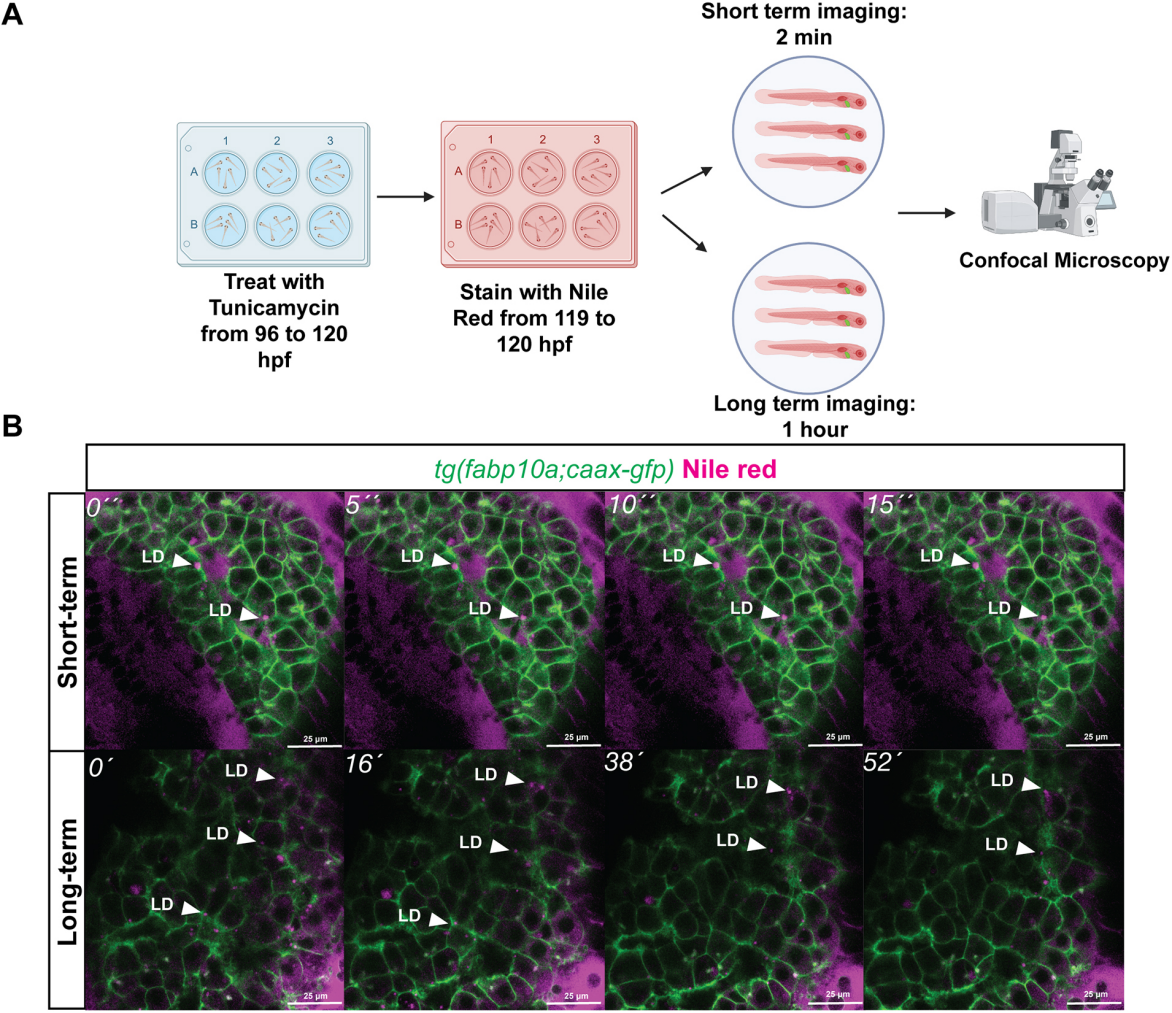
**Hepatocyte LD dynamics can be imaged with NR in live zebrafish.** (A) Schematic of short-term and long-term imaging treatment scheme using NR with TM exposure. (B) Representative stills from short-term and long-term movies of LDs in CAAX-GFP-marked larvae with NR staining at 120 hpf during TM exposure from 96 to 120 hpf. White arrowheads (LD) point to examples of LDs. Scale bars: 25 µm. The images were taken from a single *z*-section and are representative of more than six larvae from two clutches.

We took advantage of NR labelling LDs to investigate LD dynamics using timelapse imaging in 120 hpf zebrafish larvae exposed to TM. We assessed LD dynamics over a short (2 min) or long (1 h) time frame (Fig. 3A). The short timelapse images were captured every 5 s and showed that LDs are relatively static within hepatocytes (Fig. 3B and Movie 1). Over the course of 1 h, most LDs were static; however, some LDs appeared or disappeared, which could reflect their formation and utilization or the fading of NR intensity over the time course of the experiment (Fig. 3B and Movie 2). We found that the few LDs that disappeared during the course of 1 h were also detected by brightfield imaging and by EGFP-PLIN2 (Fig. S2, white arrowheads), suggesting that the dynamics observed with fluorescent dyes is not attributed to their bleaching during the time course. This demonstrates the flexibility of fluorescent lipid dyes to assess the incidence and severity of steatosis, as well as the dynamics of LDs in live animals.

We observed that the *Tg(fabp10a:EGFP-PLIN2)* reporter (Wilson et al., 2021) was dim compared to other methods of imaging LDs. To determine whether the EGFP-PLIN2^+^ LDs contained neutral lipids, we stained fasted *Tg(fabp10a:EGFP-PLIN2* larvae with NR and performed live imaging on a confocal microscope. We found that fasting caused an increase in both NR-stained and EGFP-PLIN2^+^ LDs, with the majority of LDs labelled with both (Fig. S3A). To gain more insights into differentially labelled LDs, we counted the number of LDs in which both labels were colocalized or had a single label, and measured the diameter of the LDs that were marked by *Tg(fabp10a:EGFP-PLIN2)* and those that were stained with NR in the same fasted larvae (Fig. S3A). In six larvae analysed, all EGFP-PLIN2^+^ LDs were labelled with NR, but only 85% (±6.63 s.d.) of the NR-labelled LDs were also labelled with EGFP-PLIN2. There was no difference in the size of these LDs when averaged per fish (Fig. S3B) or when comparing individual LDs labelled with NR or with EGFP in the same animal (Fig. S3C). These data suggest that either NR and PLIN2 are labelling a small subset of distinct LD pools or that NR is more sensitive than EGFP-PLIN2 at detecting LDs in zebrafish hepatocytes.

**Fig. 4.**
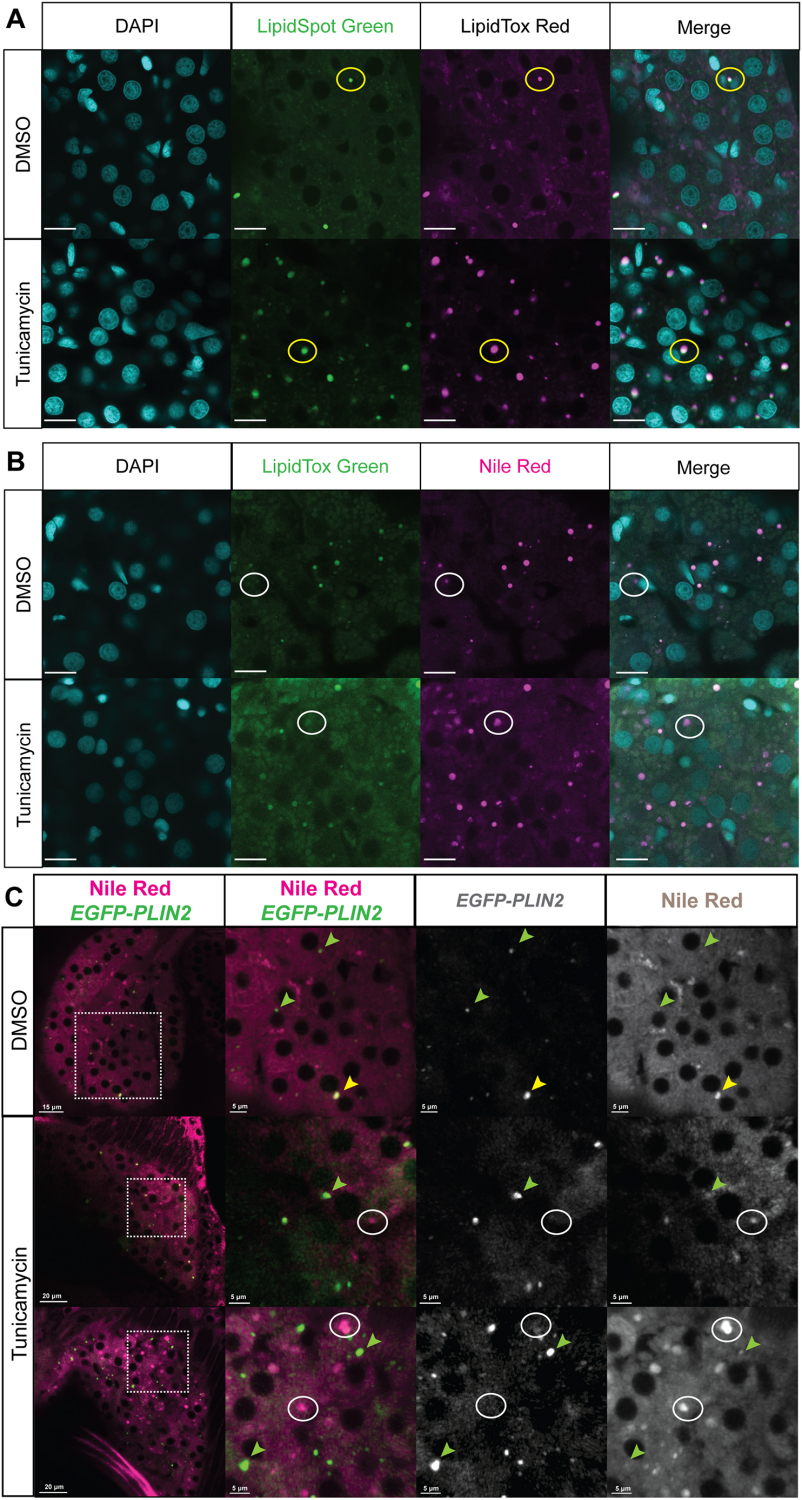
**Comparing NR to LipidTox, LipidSpot and EGFP-PLIN2 labelling of LDs in hepatocytes.** Larvae were treated with DMSO or 0.5 μg/ml TM, fixed and processed as described for fluorescent confocal microscopy to detect LDs. (A) Representative confocal images of hepatocyte nuclei (blue-DAPI) and LDs (green and magenta) co-labelled with LipidSpot and LipidTox. The 128 hpf zebrafish larvae were treated with DMSO or 0.5 μg/ml TM from 96 to 128 hpf. Scale bars: 10 μm. The yellow circles highlight an LD that is present in all images. (B) Representative confocal images of hepatocyte nuclei (blue-DAPI) and LDs (green and magenta) co-labelled with LipidTox and NR. The 128 hpf zebrafish larvae were treated with DMSO or 0.5 μg/ml TM from 96 to 128 hpf. Scale bars: 10 μm. The white circles highlight an LD that is detected by NR but not by LipidTox Green. (C) *Tg(fabp10a:EGFP-PLIN2)* larvae were treated with 0.5 µg/ml TM from 96 to 120 hpf and stained with NR for live confocal imaging. Samples were assessed for LDs labelled with NR, EGFP or both. Green arrowheads point to LDs that are detected with EGFP but not NR, white circles highlight LDs detected with NR but not EGFP, and yellow arrowheads indicated an LD that is detected with both EGFP and NR. Scale bars: 5, 15 and 20 µm as indicated on each image individually. The images were taken from a single *z*-plane and represent more than 20 larvae imaged from more than two clutches.

To complement these insights, we performed LD co-labelling studies using the fluorescent neutral dyes LipidTox Green/Red (LipidTox), LipidSpot and NR in combination. To this end, we assessed fixed larvae treated with DMSO or TM from 96 to 128 hpf. Following combinatorial staining with 4′,6-diamidino-2-phenylindole (DAPI)/LipidSpot Green/LipidTox Red, larval livers were dissected and mounted on a glass slide with a cover slip for confocal analysis. We observed that TM induced an increase in LD accumulation and that each LD stained with LipidSpot was also stained with LipidTox (Fig. 4A). Similarly, combinatorial staining with DAPI/LipidTox Green/NR revealed that all LDs were positive in both channels (Fig. 4B). Interestingly, we found that there was more autofluorescent background in the fixed tissue in the green channel, which led to lower signal to noise, and, in some cases, LDs that labelled with NR did not label with one of the green dyes (see circled LD in Fig. 4B). Similar to fasting-induced steatosis, nearly all LDs in TM-induced steatosis were labelled with both NR and EGFP-PLIN2; however, there was a small subset of LDs that were NR positive and not labelled with EGFP (circled LD in Fig. 4C). These studies demonstrate that the fluorescent LD labels investigated here label largely the same LD populations in hepatocytes.

### Quantitative assessment of LDs in fixed hepatocytes using LipidTox, LipidSpot and NR

**Fig. 5.**
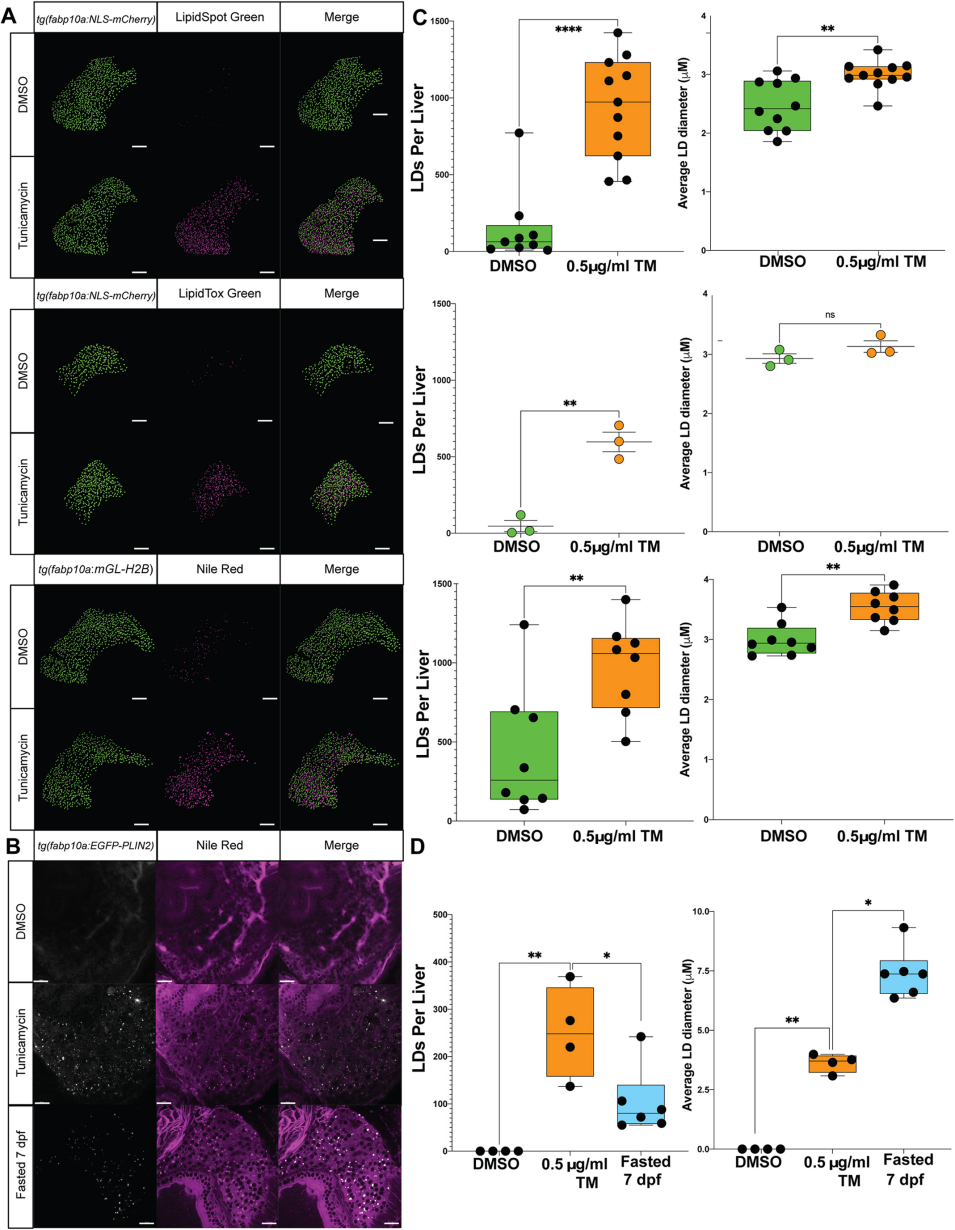
**Quantitative hepatic LD analysis using LipidTox, LipidSpot and NR.** (A) Representative Imaris-rendered confocal images of hepatocyte nuclei (green) and LDs (magenta) in zebrafish larvae treated with DMSO or 0.5 μg/ml TM from 96 to 128 hpf and stained with LipidSpot, LipidTox or NR. Transgenic lines to label hepatocyte nuclei were used in contrasting colours [i.e. *Tg(fabp10a:NLS-mCherrry)* and *Tg(fabp10a:mGL-H2B)*]*.* Scale bars: 50 μm. (B) Single confocal sections of fixed *Tg(fabp10a:EGFP-PLIN2)* larvae treated from 96 to 120 hpf with DMSO or TM, or maintained until 168 hpf as fasted samples, stained with NR. Scale bars: 20 μm. (C) Quantification of LD number and diameter in hepatocytes of zebrafish larvae stained with LipidSpot, LipidTox and NR as shown in A. Hepatic LDs for each larvae were detected to a depth of 100 μm (34 *z*-slices). Each datapoint represents a larva, and data are shown as median and interquartile range or mean and s.e.m. *n*>3. ns, not significant; **P<0.01, ****P<0.0001. Statistical analysis for LD number and size with two treatment groups was determined by unpaired two-tailed Student's *t*-test. (D) Quantification of LD number and diameter in hepatocytes of transgenic *EGFP-PLIN2* zebrafish larvae as shown in B. Hepatic LDs were detected in a single *z*-plane in the left lobe of each larvae. Each datapoint represents a larva, and data are shown as median and interquartile range. *n*>4. *P<0.05, **P<0.01. Statistical analysis for LD number and size with two treatment groups was determined by unpaired two-tailed Student's *t*-test.

Having demonstrated that all fluorescent neutral lipid dyes effectively labelled the same LDs, we were motivated to use a quantitative approach to measure LD abundance and size following confocal or multiphoton microscopy. We developed an analysis pipeline that takes advantage of Imaris imaging software to render and quantify the LDs in combination with transgenic liver reporters [*Tg(fabp10a:NLS-mCherry)* and *Tg(fabp10a:mGL-H2B)*] (Fig. S4). Using this approach, we imaged and quantified LD abundance and size in DMSO- and TM-treated larvae at 128 hpf following staining with LipidSpot, LipidTox and NR (Fig. 5A,B). We found that each of these dyes detected a comparable number of LDs in response to TM, ranging from 600 to 1000 LDs per liver (Fig. 5C,D). The number of LDs present in DMSO-treated control livers averaged ∼0-200 LDs per liver, with some outliers that were more lipid laden. With respect to the LD diameter, we determined that the average LD size increased from 2.5-3 µm in DMSO controls to ∼3-3.5 µm upon TM exposure (Fig. 5C,D). Interestingly, LipidSpot and NR exhibited slightly higher sensitivity at detecting LDs than LipidTox, especially in deeper *z*-sections of tissue. We also determined that different inducers of steatosis led to distinct LD phenotypes, with fasting-induced steatosis leading to fewer LDs with a larger diameter (Fig. 5B,D). Together, these studies illustrate the features that can be ascertained from quantitative Imaris analysis of fluorescently labelled LDs.

## DISCUSSION

This comparative study of the methods used to visualize LDs in the hepatocytes of zebrafish larvae includes transgenic LD reporters, the colorimetric dye ORO, and the fluorescent dyes NR, LipidTox and LipidSpot. We provide detailed protocols for sample preparation, staining, imaging and image analysis, and summarize the properties of each approach in Table 2. The methods presented offer a range of cellular resolution detection methods for hepatic LDs, utilizing both low-power light microscopy and advanced confocal fluorescent microscopy in live and fixed animals. We anticipate that these methods will be useful for future studies on hepatocyte LDs in zebrafish, a topic relevant to metabolic dysfunction, nutrient surplus, diabetes and toxicant exposures, all of which cause steatosis.

Since the first study nearly two decades ago describing the use of ORO to detect lipids in zebrafish (Sadler et al., 2005), this lipophilic stain, along with histopathological assessment of H&E-stained sections, has become a standard approach to assess lipid in the liver and other structures in zebrafish (Howarth et al., 2013; Yoganantharjah et al., 2017; Zhou et al., 2015)*.* These techniques are used clinically to diagnose steatosis and provide a low-cost and easily accessible approach for quantifying the incidence of steatosis in a large population of zebrafish larvae. The limitation of this approach is that ORO staining is less sensitive than fluorescent dyes and can be variable between researchers, as quantification is limited to binary scoring of the presence or absence of steatosis in fixed samples. Therefore, for enhanced quantitative analysis of LDs, fluorescent dyes are preferable.

The main advantage of using fluorescent dyes to visualize LDs is the ability to acquire images at cellular resolution. Fluorescent dyes can be combined with genetically encoded reporters of cellular structures (cell membranes or nuclei), and they can be imaged in fixed or live tissue, which is a distinct advantage of the zebrafish model. Staining with fluorescent dyes is simple, requiring few reagents for sample preparation. These dyes provide more nuanced data on the formation, distribution and function of LDs in hepatocytes. For instance, one of the fluorescent neutral LD dyes can be used in conjunction with another cellular marker for an organelle, cellular structure or protein of interest. As examples, *in vivo* imaging of LDs with a membrane marker (Figs 1-3) or the nucleus (Figs 4 and 5) can provide information about the localization and distribution of LDs relative to these cellular structures. In the future, we anticipate that researchers will utilize other fluorescent dyes in combination with the neutral LD fluorescent stains presented in this study. One limitation of the use of lipid dyes is that they require sophisticated and costly microscopes as well as the expertise to use them, which can limit the applicability to investigators with easy and affordable access to suitable microscopy facilities. We were unable to reliably detect LD accumulation in the liver using the stereomicroscopes available in our laboratories.

NR is a versatile fluorescent probe that can detect neutral LDs and phospholipids via fluorescent imaging and flow cytometry (Greenspan et al., 1985). Our study demonstrates that NR can also be used to visualize hepatic LDs *in vivo* using confocal and multiphoton imaging techniques. Previous research has utilized NR dye to investigate adipose tissue biology in zebrafish (Bergman et al., 2001; Zimmermann et al., 2004) and to describe the development of this tissue through juvenile stages (Flynn et al., 2009; Minchin and Rawls, 2017; Minchin et al., 2018)*.* Recently, transgenic LD reporter lines have been developed that rely on fusion of fluorescent proteins to perilipins (PLIN2 or PLIN3) that decorate the LDs (Lumaquin et al., 2021; Wilson et al., 2021). This innovation allows for live imaging of fluorescent LDs in tissue without the need for lipid staining. One powerful advantage of *in vivo* imaging is the opportunity to track LD dynamics over time, and we demonstrated that LDs are stable up to a 1 h imaging time course. Collectively, these approaches highlight the advantages of using fluorescent markers to study the role of LDs at both cellular and organismal levels.

Imaging of fixed tissue *in situ* offers flexibility, with fewer time constraints and the ability to process and image a larger number of samples. We determined the compatibility of NR, LipidTox and LipidSpot staining in fixed zebrafish larvae tissue. It should be noted that a caveat when using fluorescent imaging is that fixation can generate autofluorescence. However, the signal generated from NR, LipidTox and LipidSpot is significantly higher than that from autofluorescence, and we have not found this to compromise image quality or quantification. Our studies are the first, to our knowledge, to report the use of LipidSpot to detect hepatic LDs in zebrafish larvae*.* Importantly, our direct comparison of NR, LipidSpot and LipidTox showed that, although these dyes all have the capacity to stain LDs, NR appeared to have the highest sensitivity in detecting LDs in zebrafish hepatocytes.

Alcohol abuse, diabetes, nutrient surplus and toxicant exposures have all contributed to the rise in metabolic liver disease worldwide (Alswat et al., 2018; Anstee et al., 2019; Llovet et al., 2021). This worrying trend may result in a rise in severe hepatic conditions, such as steatohepatitis, cirrhosis and liver cancer. Understanding how organisms regulate cellular metabolism is crucial for comprehending the development of MASLD and ALD. Zebrafish larvae serve as a valuable model for studying liver disease pathogenesis owing to their significant anatomical and metabolic pathway similarities to those of humans (Holtta-Vuori et al., 2010; Kinkel and Prince, 2009; Zang et al., 2018). Our refined LD staining techniques offer a straightforward, yet powerful, approach for examining LDs *in vivo*. We hope that the methods presented will be a valuable resource for future work in this emerging area.

## MATERIALS AND METHODS

### Zebrafish husbandry and culture conditions

Zebrafish (*Danio rerio*) husbandry and care was conducted according to institutional Australian Animal Experimentation Ethics Committee (AEEC) guidelines (Approval E580, E666, E534) and the New York University Abu Dhabi (NYUAD) for Animal Care and Use Committee (IACUC) Committee (protocol number 22-0003A2). The zebrafish lines *Tg(fabp10a:NLSmCherry)* (Mudbhary et al., 2014) and *Tg(fabp10a:mGreenLantern-H2B)* (Tan et al., 2024) were used to mark the nucleus of hepatocytes. *Tg(fabp10a:CAAX-EGFP)* (Nussbaum et al., 2013) was used to mark the cell membrane of hepatocytes. *Tg(fabp10a:EGFP-PLIN2)* (Wilson et al., 2021) was used to mark hepatocyte LDs. Embryos were generated by natural spawning of groups of adult zebrafish. Clutch siblings were used as controls. All embryos were raised and maintained in E3 medium (5 mM NaCl, 0.17 mM KCl, 0.33 mM CaCl_2_, 0.33 mM MgSO_4_ in Milli-Q water) at 28°C on a light–dark cycle (14 h:10 h) and maintained using standard culture protocols (Magnani et al., 2024; Ramdas Nair et al., 2021). All experiments were conducted at larval stage, so the sex of the organism could not be confirmed.

### Chemical treatment of zebrafish larvae

At 96 hpf, larvae were treated with either 0.5 μg/ml TM, 95% mixture of congeners (Sigma-Aldrich, T7765) or 350 mM ethanol until 120 hpf in a six-well plate stored in the 28°C bench incubator. Control larvae were allocated and treated with an equal volume of DMSO (Sigma-Aldrich) as vehicle controls, where the final concentration of DMSO in the medium did not exceed 0.1% and was at the equivalent concentration applied in the experimental group. Fasted animals were maintained no later than 168 hpf without nutritional supplementation.

### ORO staining

ORO staining of larvae fixed in 4% paraformaldehyde (PFA) in phosphate buffered saline (PBS) was carried out as described previously (Cinaroglu et al., 2011; Passeri et al., 2009). In short, fixed larvae were washed with PBS, infiltrated with a graded series of propylene glycol then transferred to 0.5% ORO (Sigma-Aldrich, O0625) in 100% propylene glycol overnight at room temperature. They were de-stained in 100% then 85% and 40% propylene glycol to PBS and then stored indefinitely at room temperature in 80% glycerol/PBS. ORO-stained larvae were imaged using light microscopy on a Nikon SMZ25 Stereoscope. Larvae were scored as positive for steatosis if they had three or more LDs in hepatocytes in the entire liver.

### H&E staining

Zebrafish larvae were fixed in 4% PFA and embedded in agarose larval arrays. After paraffin embedding and serial sectioning, slides were stained with H&E using a previously published protocol (Vaidyanathan et al., 2022).

### NR staining and live imaging

NR stock (Sigma-Aldrich, 72485-100MG) was prepared at 1 mg/ml in acetone or methanol. Zebrafish larvae cultured in six-well plates were stained at 119 hpf just prior to imaging by adding NR to the wells at a final concentration of 500 ng/ml and incubated for 1 h in the incubator at 28°C, wrapped in aluminium foil. For live imaging, the NR-containing medium was replaced with E3 medium lacking NR and containing 0.16 g/l tricaine. For fixed samples, following NR staining, larvae were fixed with 4% PFA overnight at 4°C, washed twice with 2 ml 1× PBS and processed in the same fashion as for live samples for confocal imaging.

Live or fixed larvae were transferred to a tissue culture dish with cover glass bottom using a transfer pipette and embedded in 1% low-melt agarose as described (Magnani et al., 2024). Using a thin paintbrush or a hairloop, the larvae were pushed to the bottom of the dish so that the left-lateral side of the larvae was at the bottom to enable imaging on the inverted microscope. The dish was left undisturbed in the dark for 15 min or until the agarose solidified/gel stiffened. Prior to live imaging, the dish was filled with E3 water and tricaine mix, (0.08 g/l) and for fixed larvae it was filled with PBS. NR-stained larvae were imaged on a Leica SP8 3× STED laser confocal microscope using a 63× water objective lens. To compare the LDs stained with NR at different wavelengths, we used excitation wavelengths of 450-500 nm and 515-600 nm with emission mode at 528 nm and 590 nm, respectively. For short- and long-term live imaging, NR-labelled structures were imaged with a temporal resolution of 5 s for 2 min for short-term imaging and every 120 s for 2 h for long-term imaging.

For comparison of NR and ORO staining, live larvae were imaged for NR as described above, scored for steatosis, then removed into individual 2 ml round-bottomed tubes to be stained with ORO and scored for steatosis as described above. All fish in a sample were scored as positive if they had two or more LDs per left liver lobe or negative if they had one or zero droplets, and the incidence was the percentage of positive larvae per clutch. Steatosis severity was assessed by counting the number of LDs divided by the area on a single confocal section through the middle of the left liver lobe and expressed as number of LDs/µm^2^.

### LipidTox and LipidSpot staining

Zebrafish larvae (128 hpf) were fixed in 4% PFA and left rocking at 4°C overnight. The next day, the larvae were washed twice with PBS with Tween 20 (PBST). The remaining PBST was aspirated, and 1.5 μl of either LipidSpot (Biotium, 70065-T), HSC LipidTox Green Neutral Lipid (Thermo Fisher Scientific, H34475), or HSC LipidTox Red Neutral Lipid (Thermo Fisher Scientific, H34476) was added to 1.5 ml Milli-Q water containing the larvae. The tubes were immediately wrapped with aluminium foil and left to rock overnight at room temperature. The following day, stained larvae were mounted in larval agarose arrays, sealed with 1% low-melt agarose and imaged under an Olympus FVMPE-RS multiphoton microscope. LD staining in the liver was visualized and quantified using FIJI and Imaris.

### Dye colocalization analysis

Fixed and stained tissue of 128 hpf larvae were mounted with 1% low-melt agarose and Fluoromount-G^TM^ mounting medium in a single cavity glass slide 1-1.2 mm thick to 26×76 mm super-grade microscope slides sealed with a coverslip for confocal microscopy. Imaging was performed using an Olympus FV3000 confocal microscope, on Galvano [1 frame/s (fps)] with silicone oil immersion 60×/1.30 NA lens. *Z*-stacks were taken with a 1 μm step size. Lasers used were 405 nm, 488 nm and 561 nm to detect DAPI (nucleus), green channel (LipidSpot or LipidTox) and red channel (LipidTox or NR), respectively (Table 1). Laser intensities were kept low and consistent to limit background fluorescence. *Z*-stacks were processed and analysed using FIJI or Imaris.

### Multiphoton microscopy

Fixed 128 hpf zebrafish larvae tissue were arrayed on a specialized agarose mould (Sabaliauskas et al., 2006) using a thinned out paintbrush or hairloop. Larvae were embedded with the head of the larvae pointing to the left and the left-hand side of the liver facing up. The fluorescent lipid probes used have a variety of excitation and emission wavelengths (summarized in Table 1). Multiphoton microscopy was used to examine whole-liver and respective LD stains (NR, LipidSpot, or LipidTox). Imaging was performed using the Olympus FVMPE-RS (C4.8) multiphoton microscope with a 25×/1.05 NA objective lens and Olympus FV30-SW software, on Galvano (1 fps) and Resonance (30 fps) scanners in 16-bit mode, with water immersion 25×/1.05 NA, XLPLN25xWM objective lens and Olympus FV30-SW software. *Z*-stacks were taken at 3 μm step sizes, with a depth of 100 μm. Multiphoton excitation was provided by Insight Deepsee (680-1300 nm) and Maitai Deepsee (690 nm-1040 nm) lasers. Excitation wavelengths for DAPI (nucleus), LipidTox Green (LD) and LipidSpot (LD), NR, NLS-mCherry (hepatocyte nucleus) and LipidTox Red (LD) were 740 nm, 820 nm, 950 nm, 850 nm, and 1100 nm, respectively. Emissions for DAPI (nucleus), LipidTox Green (LD) and LipidSpot (LD), NR, NLS-mCherry (hepatocyte nucleus) and LipidTox Red (LD) were all detected at 460 nm, 510 nm, 510 nm, 550 nm and 610 nm respectively. Laser powers were kept low and consistent to limit background fluorescence. *Z*-stacks were processed and analysed using Imaris.

### Image analysis

Imaris v9.3 (Imaris) was used to quantify LD number and diameter in the context of transgenic lines demarcating hepatocyte nuclei [*Tg(fabp10a:NLS-mCherry)* and *Tg*(*fabp10a:mGreenLantern-H2B*)]. In Imaris, the functions ‘Spot’ and ‘Surface’ were used for quantifications. Both functions rely on the maximum-intensity projection mode and involve multi-step processes that are illustrated in Fig. S4. All the images acquired from the multiphoton microscope were converted to IMS format prior to Imaris processing. The ‘Spot’ function was used to measure the number of hepatocytes, number of LDs and average diameter of LDs. In each case, the pipeline is the same with minor differences. The IMS format image was opened, and the ‘Spot’ function was selected. First, the region of interest was selected to specify the X, Y and Z dimensions that only contain the liver. The Z dimension was kept constant for every analysis (0-100 μm) in each experiment (NR, LipidSpot and LipidTox). For the measurement of LD diameter, the ‘different spot sizes (region growing)’ function was selected. The channel for the appropriate staining was then selected. Next, the estimated XY diameter section was filled, with every hepatocyte nucleus and LD measurement 5 μm and 3 μm, respectively. The thresholding was applied using the ‘background subtraction’ function. The threshold of selection was adjusted to only include authentic signals in the liver. The authentic signal is defined as spherical structures with the appropriate size and location (in the liver area). Then, the ‘absolute intensity’ option was selected. Only for the LD diameter measurement, the ‘Spot region’ threshold was adjusted to depict the most accurate measurement. The ‘Region Volume’ option was also selected. Finally, after the processing was complete, quality control was performed by inspecting the rendered structures to remove any potential background or false signals. The number of LDs was normalized per liver. Quantifications were exported as Microsoft Excel files. The representative images of rendered structures were pseudo-coloured for ease of visualization and exported using the ‘Export’ function.

### Quantification and statistical analysis

All experiments were carried out on at least three larvae from at least three clutches per condition, with exact numbers indicated either on the figure or in the figure legends. Statistical analysis was carried using GraphPad Prism 9 using one- or two-way ANOVA or unpaired two-tailed Student's *t*-test, as indicated.

### Digital image processing

Schematic workflows showing sample collection and image acquisition were created using BioRender.

## Supplementary Material

10.1242/dmm.050786_sup1 Supplementary information
