# Supplementary material for: Optimized methods to image hepatic lipid droplets in zebrafish larvae
Source: Dis Model Mech. 2024 Nov 25;17(11):dmm050786. doi: 10.1242/dmm.050786 (PMC11625896; doi:10.1242/dmm.050786)
Supplement: Supplementary information [file dmm-17-050786-s1.pdf]

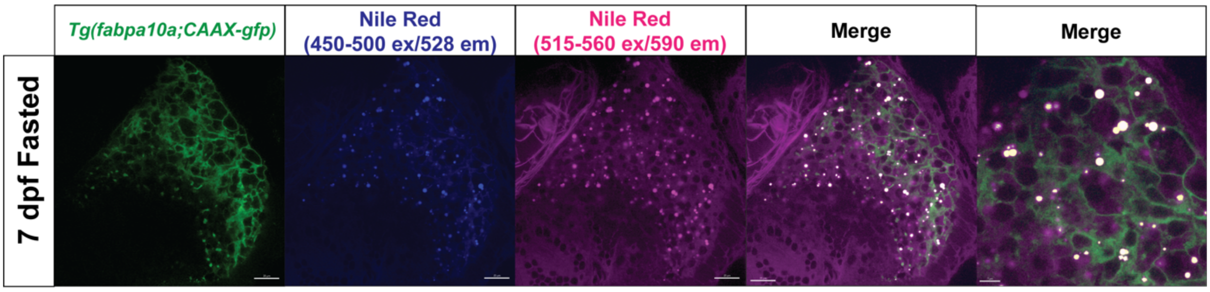

Fig. S1. Detection of LDs and phospholipids using NR at distinct excitation and emission wavelengths. Larvae that were fasted until 7 dpf were fixed and stained with NR as described. The same larvae were imaged using a 515-560 excitation/590 emission or 450-500 excitation/528 emission to image LDs and LDs plus phospholipids, respectively.

Disease Models & Mechanisms • Supplementary information

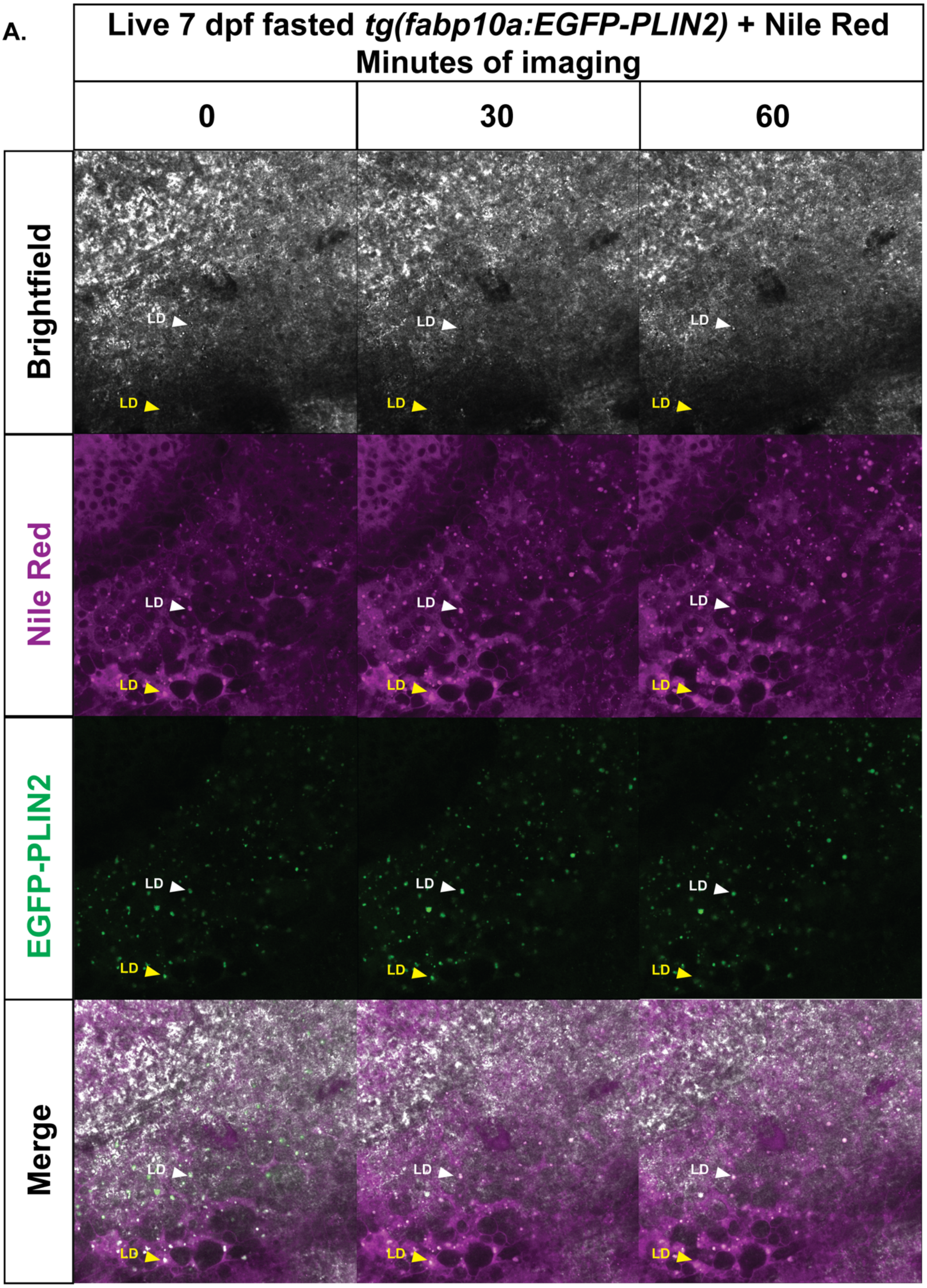

Fig. S2. NR and EGFP-PLIN2 label LDs in zebrafish hepatocytes. *Tg(fabp10a:PLIN2-EGFP)* larvae were fasted until 7 dpf and stained with NR for time lapse confocal imaging of a single optical section over 60 minutes. Samples were assessed for colocalization of NR and GFP on LDs. A static LD is labelled with white arrowhead and a dynamic LD is labelled with a yellow arrowhead. All imaging for these still were conducted on a single z-stack.

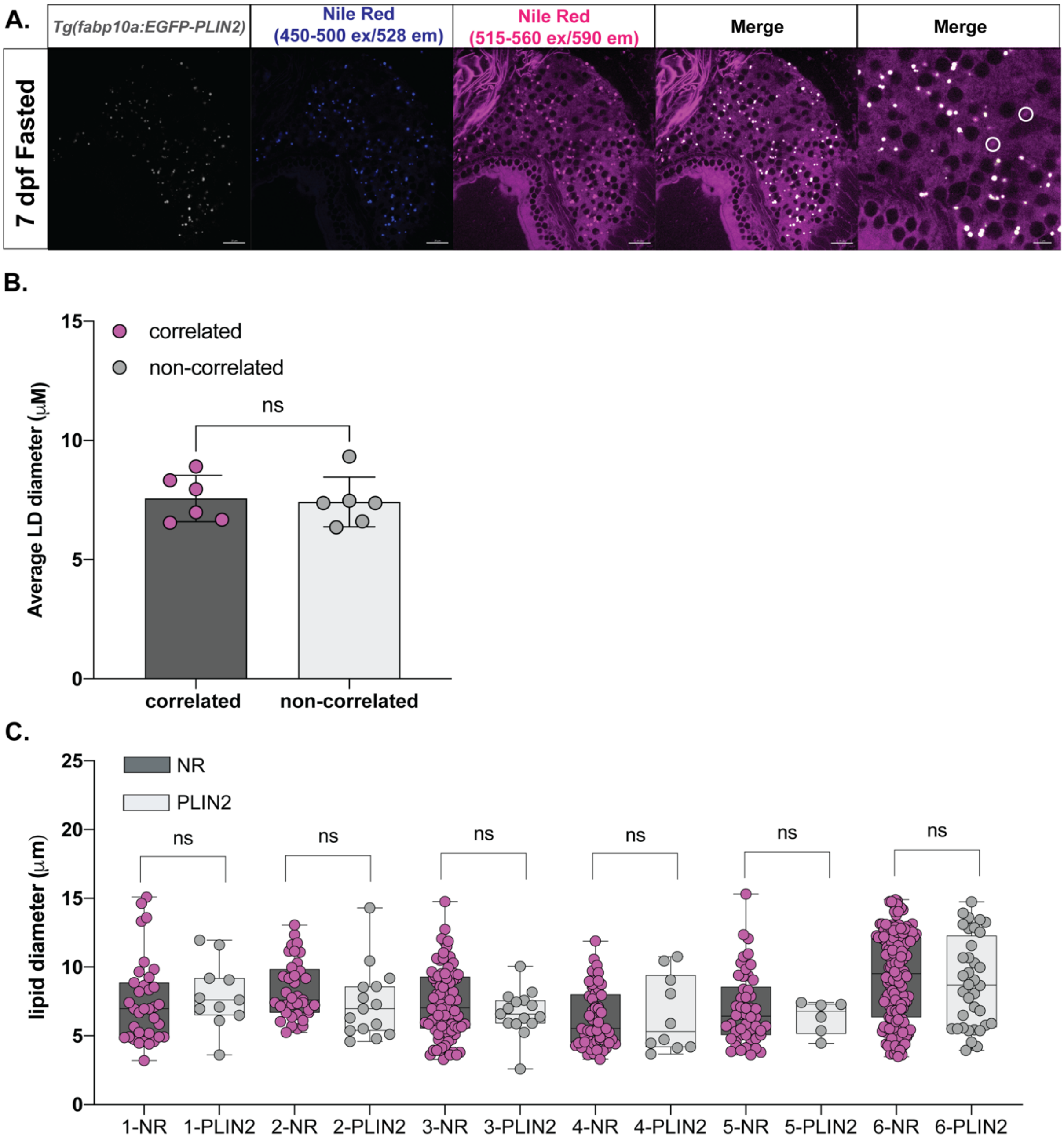

Fig. S3. NR and PLIN2 label distinct sub-populations of LDs.

Tg(fabp10a:EGFP-PLIN2) fasted larvae were stained with NR and imaged using excitation/ emission wavelengths to detect NR stained LDs (blue) and LDs plus phospholipids (magenta). The overlay image shows that nearly all LDs are labelled with both markers, but there are a few that are NR positive and GFP negative (white circles). Scale bars represent 15 μm. **B.** The average diameter per liver of the LDs that are labelled with both NR and EGFP (correlated) as well as those that are labelled with only NR (non-correlated). **C**. The diameter of individual LDs marked with NR or with EGFP was measured in 6 livers. ns=not significant.

Disease Models & Mechanisms • Supplementary information

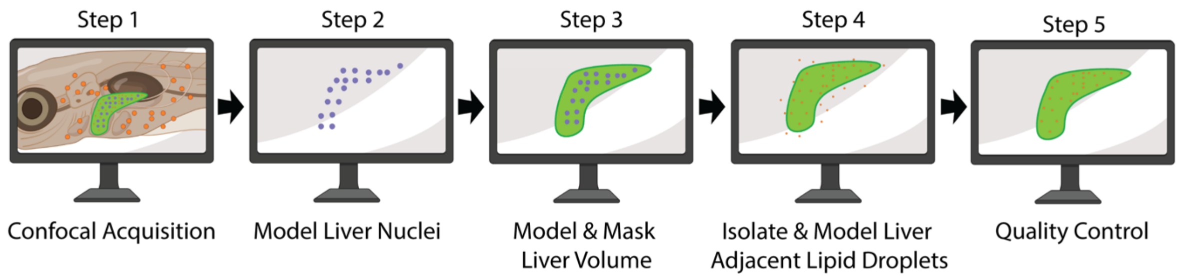

Fig. S4. Schematic of the Imaris pipeline used to quantify hepatic LDs. Step 1: Acquire images on the multiphoton microscope. Step 2: Using the spot function of Imaris, fluorescence microscopy data of liver nuclei fluorophores is used to model liver nuclei. A 3D (XYZ) ROI is selected such that contains that liver and with a Z-dimension restricted to 100 microns (34 z-slices) to maintain consistency. Estimated XY diameter is set to 5 microns, and a brightness threshold is manually set using background adjustment. This is to ensure background fluorescence can be discriminated from true fluorescence. Step 3: The fluorescence channel is then used to create a pseudo-volume of the liver utilising the surfaces function. A mask is deployed to produce a subset of the fluorescence information that is restricted to the liver volume. Step 4: Using this subset, LDs contained within (and slightly adjacent) to the liver can be modelled. This enables for a more specific ROI than the 3D cube Imaris can provide. We set the spot function to “region growing” to allow for varying spot size. Estimated XY diameter is set to 3 microns, with brightness thresholding performed as above. Step 5: To ensure the variable spot size is accurate, the ‘Region volume’ thresholding is verified manually. This step ensures that erroneously identified LDs are identified and removed from the analysis.

Disease Models & Mechanisms • Supplementary information

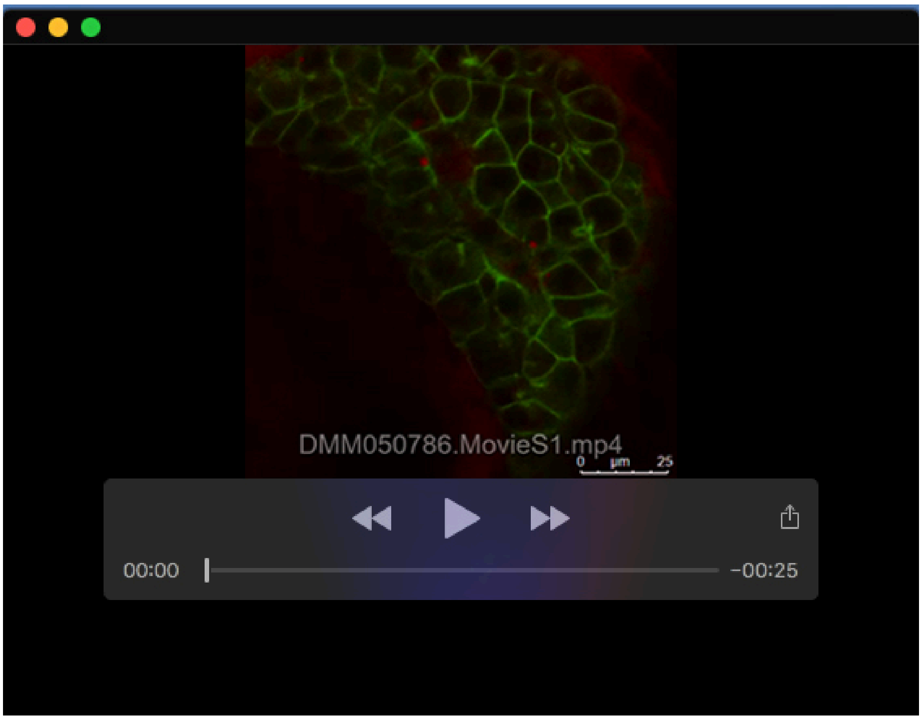

Movie 1. Minimal LD movement in zebrafish hepatocytes
Tg(fabp10a:CAAX-EGFP) larvae treated with 0.5 µg/ mL TM from 96-120 hpf were stained with NR and imaged on a single confocal plane for 2 minutes with 5 second intervals using 63x water immersion lens. LDs are static during this image capture period.

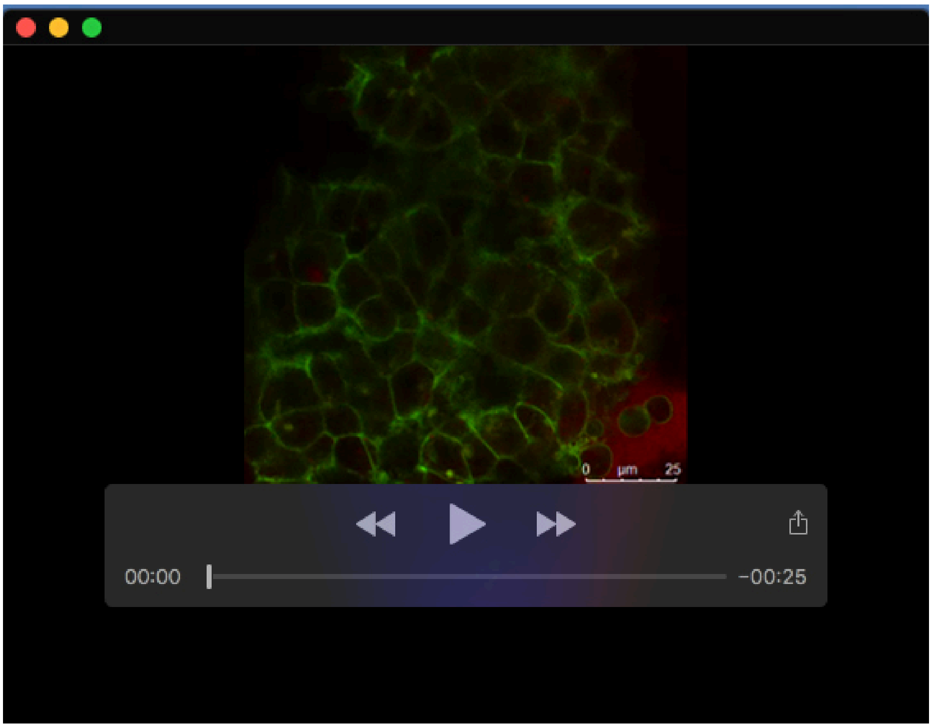

Movie 2. LD dynamics during long term imaging of zebrafish hepatocytes
Tg(fabp10a:CAAX-EGFP) larvae treated with 0.5 µg/mL TM from 96-120 hpf were stained with NR and imaged live for 1 hour with image capture 120 second intervals using 63x water immersion lens. One LD is observed to disappear while the other LDs are static during this image capture period.
